# COVID-19 Vaccine Acceptance Among Dental Professionals Based on Employment Status During the Pandemic

**DOI:** 10.3389/fmed.2021.618403

**Published:** 2021-02-09

**Authors:** Asaf Zigron, Amiel A. Dror, Nicole G. Morozov, Tali Shani, Tharwat Haj Khalil, Netanel Eisenbach, Doaa Rayan, Amani Daoud, Fares Kablan, Hesham Marei, Eyal Sela, Samer Srouji

**Affiliations:** ^1^Oral and Maxillofacial Department, Galilee Medical Center, Nahariya, Israel; ^2^Department of Otolaryngology, Head and Neck Surgery, Galilee Medical Center, Nahariya, Israel; ^3^The Azrieli Faculty of Medicine, Bar-Ilan University, Safed, Israel; ^4^Sackler Faculty of Medicine, Tel Aviv University, Tel Aviv, Israel; ^5^College of Dentistry, Gulf Medical University (GMU), Ajman, United Arab Emirates

**Keywords:** COVID-19, vaccine hesistancy, unemployment, SARS-CoV-2, vaccine

## Abstract

The COVID-19 pandemic spread rapidly across the globe, leading governments to impose prolonged lockdowns on both movement and commerce. Although lockdowns decrease the rates of novel infections, they can have devastating consequences on the economy and employment levels. One of the most severely affected sectors during this crisis has been dental medicine. Dental professionals are uniquely exposed to environments with high levels of occupational hazards, conferring additional risks of viral exposure and transmission. We analyzed 506 anonymous questionnaires completed by dentists and residents regarding acceptance of a future potential SARS-CoV-2 vaccine. Our results demonstrate a statistically significant correlation between the individual's unemployment rate and their willingness to inoculate with a SARS-CoV-2 vaccine when it becomes available. This information can be used to predict trends of vaccine acceptance or rejection based on economic burden during the COVID-19 pandemic by different sectors as part of the preparedness toward global vaccination programs.

## Introduction

Beginning late December 2019, the SARS-CoV-2 coronavirus spread rapidly worldwide, leaving in its wake devastating impacts on human mortality, social behaviors, economies, and healthcare systems. Long lockdown periods imposed by governments led to substantial operational obstructions of vast arrays of economic sectors including cultural institutions, restaurants, tourism, and travel, leading inevitably to soaring unemployment rates. Experts estimate that what has been seen so far is merely the tip of the iceberg, and only in many years will experts be able to assess the final consequences of the COVID-19 pandemic on the global economy. The International Labour Organization (ILO) announced on 15 June that 32% of the world's workers were living in countries with lockdown-related workplace closures for all but essential occupations (1). The ILO has coined the term “lockdown generation” to refer to youths particularly impacted by the global market depression which could, in its estimates, last a decade or longer (2).

One of the most acutely affected sectors has been dentistry and its related residencies. Dental professionals are uniquely exposed to environments with high levels of occupational hazards due to aerosols and oral fluids, conferring additional risks of viral exposure and transmission (3, 4). During the lockdown period in Israel between March 17th and April 19th, all elective procedures were postponed due to government order. The only treatments offered during the pandemic were those indicated for trauma, pain, head and neck infections, and malignant tumors, primarily performed by Oral and Maxillofacial (OMFS) surgeons and oral medicine specialists at hospitals. Minor first aid treatments have been performed in limited numbers of public and private dental clinics.

As an integral part of the fight against the COVID-19 pandemic, the World Health Organization (WHO) led the global effort in prevention, diagnosis, and treatment against this elusive pathogen. A simultaneously sustained race to discover an effective vaccine by more than 90 vaccine companies and over 100 countries is underway worldwide (5). Development of a vaccine appears to be the most promising means of restoring normalcy to civilian life and initiating economic rehabilitation. Nevertheless, SARS-Cov-2 vaccine's availability does not symbolize the end of the pandemic due to ongoing vaccine hesitancy and anti-vaccination movements (6). The WHO declared in 2019 that vaccine hesitancy is one of ten major threats to global health (7); echoing these fears, one recent study found 76% of SARS-Cov2 vaccine hesitancy is due to safety concerns (8).

The aim of our study was to evaluate the current vaccination compliance rate in correlation to unemployment among Israeli dentists, dental residents, and oral medicine specialists. The term “unemployment” in this paper refers to individuals willing but unable to work due to government-imposed workplace restrictions. The dental field can potentially reflect attitudes among other sectors, leading to a greater understanding of sentiments toward the vaccine and the development of plans to combat vaccine hesitancy. We distributed a multicenter anonymous questionnaire across Israel, asking if the dentist, resident, or specialist would agree to receive a SARS-CoV-2 vaccine once available. We analyzed the 506 responses based on occupation status in the lockdown period and willingness to vaccinate against SARS-Cov-2. All questionnaires were filled out during the mandatory quarantine period in Israel. We hypothesized that a higher rate of vaccine compliance would be observed among those who were unemployed at the time of the COVID-19 crisis.

## Study Design

### Methods

As previously described (8), the study design and protocol were approved by the Research Ethics Committee of Galilee Medical Center and the web-based survey followed the American Association for Public Opinion Research (AAPOR) reporting guidelines. The survey was distributed during the lockdown period in Israel (March- April 2020) and data was collected from dentists, dental residents, and oral medicine specialists. The survey was distributed electronically via Qualtrics health care professionals via social networks and professional forums. Before filling out the survey questionnaire, each responder had to agree and sign for electronic informed consent, which was presented at the survey's introductory web page; additionally, the survey was anonymous to ensure the confidentiality of information. The survey consisted of a series of multi-choice questions and respondents were allowed to terminate the survey at any time point.

### Data Collection

As previously described (8), demographic data were self-reported by the participants including gender (male or female), age (18–25, 26–30, 31–40, 41–50, 51–60, or >60 years) and geographic location. Specific questions asked whether the respondent is a specialist (e.g., oral medicine, orthodontics, OMFS, etc.) or general practitioner, whether he or she is a resident or practicing doctor, and place of work (hospital or private clinic). Questions regarding the status of employment during the COVID-19 crisis were included (e.g., working as usual, temporary unemployment, or lost job) though questions of religion or ethnicity were excluded. Participants were asked if they are willing to accept a future COVID-19 vaccination when it becomes available. To assess the willingness to inoculate with future SARS-CoV2 vaccine and correlation to unemployment status among dentistry healthcare sectors, we performed Chi-square and correlation tests in prism 8 software (Graphpad CA). Though the survey was not pretested, the number of respondents was sufficient to be statistically validated before initiating research.

## Results

**Figure 1:**
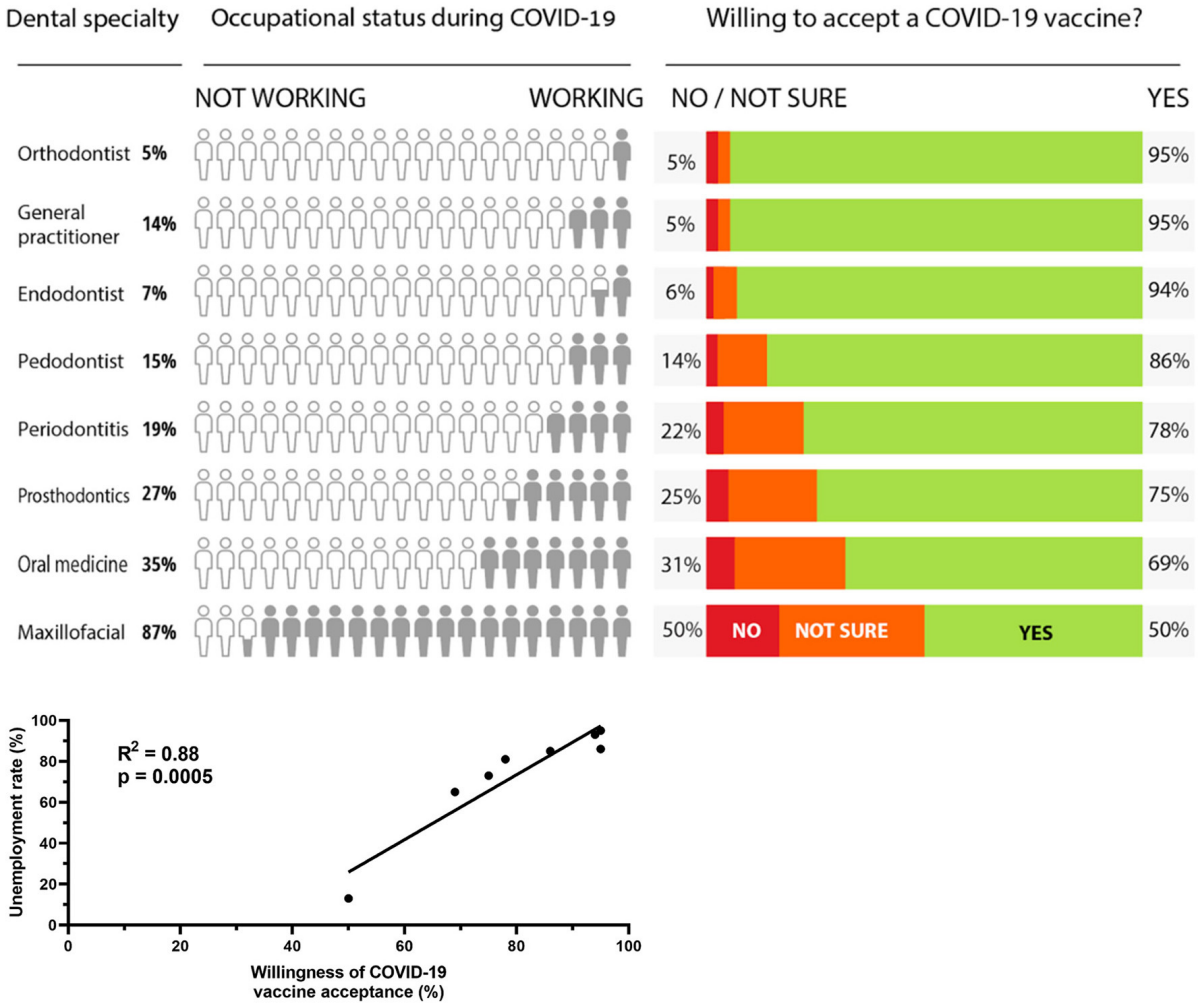
We estimated the strength of the association between willingness of COVID-19 vaccine acceptance and unemployment rate by using Pearson's correlation coefficient (*r* = 0.9414). We used GraphPad Prism version 8.2.1 (GraphPad Software, La Jolla, CA, USA) for correlation analyses.

According to our survey, the results demonstrate a statistically significant correlation between an individual's unemployment rate and their willingness to inoculate with the novel SARS-CoV-2 vaccine (Figure 1). An increase in the unemployment rate within the dental sector coincides with a rise in willingness for a SARS-CoV-2 vaccine while the converse, in which a decrease in unemployment results in a decreased willingness for inoculation, also occurs. While 50% (maxillofacial surgeons) of dental professionals are willing to receive a vaccine, over 50% of respondents for every other specialty are willing to be inoculated. The overall rate of acceptance for a COVID-19 vaccine, according to our survey, is 85%.

Of 506 respondents (57% females), 267 (53%) work as general practitioners; among them, 86% were unemployed during the lockdown period. One hundred and seven residents (21%) and 132 specialists (26%) responded to the questionnaire with variable unemployment rates depending on residency type. The mean respondent age was 36.3 for both sexes. The highest employment rate is observed among OMFS residents and specialists, 87% of whom continued to work during the COVID-19 lockdowns, work which can be attributed to mainly hospital-based operations. An interesting finding consistent with our observed trends finding is that the willingness to accept a COVID-19 vaccine among OMFS surgeons (residents and specialists) is the lowest (50%, *r* = 0.9414).

## Discussion

The current pandemic has brought about a new crisis in healthcare as the number of COVID-19-positive patients has risen dramatically worldwide. The signs and symptoms of SARS-CoV-2 were studied in tandem with the rapid development of a vaccine against this fatal disease. Global, sustained efforts have been undertaken by the WHO to limit the spread of infection and improve treatment protocols to decrease morbidity and mortality. The actions to contain the pandemic purchased time for development of effective and safe COVID-19 vaccines which as of the writing of this article are unreleased. Based on recently published research on the explanations for vaccine hesitancy, we hypothesized a correlation exists between unemployment and willingness to accept a SARS-CoV-2 vaccine. Like individuals working directly with COVID-19 patients, those who lost their jobs during the crisis may more acutely feel the impact of lockdowns and economic closures (8).

The future SARS-CoV-2 vaccine is considered by many countries as the last hope for protecting the population and economy against COVID-19 (9). Successful vaccines rely on high vaccine uptake among populations. However, recent evidence predicts an unsatisfactory acceptance rate of a COVID-19 vaccine in the general population. A survey among the general population in the United States in June 2020 suggests that only 50% of Americans are willing to get vaccinated once the vaccine is available (10). A survey in Europe, conducted in April 2020, predicts only a ~70% acceptance rate for the future COVID-19 vaccine (11). A cross-sectional questionnaire of Israeli hospital workers during the lockdown recorded that 94% of healthcare workers within respiratory wards and COVID-19 departments plan to get a COVID-19 vaccine while, surprisingly, only 61% of the nurses working in non-COVID-19 departments declare they will accept the future vaccine (12). This number is especially low when compared to the predicted acceptance rate of 75% among the general population in the same study. The date of survey enrollment may influence the public's opinion based on the extent of local health authority control on disease progression, the number of severely ill patients, and casualties (12).

Research on dentists' and dental students' acceptance of vaccinations has indicated incomplete compliance with recommended vaccinations across several countries. Research in Germany indicates there is low influenza vaccination compliance among German dental healthcare workers (13). Despite a robust understanding of the benefits of vaccination, ~20% of Italian dental healthcare workers were not up to date on recommended vaccinations (14). Understanding the underlying concerns behind vaccine hesitancy, particularly among professionals who are highly educated as to the benefits of vaccination, may provide areas of approach and education for public, and global health experts.

Although restrictions and lockdown have been eased in many countries, the ongoing COVID-19 pandemic continues to be a significant burden on the economy and many unemployed individuals are trying, unsuccessfully, to return to the labor force. Because unemployment affects not only the economy but also has direct effects on psychological and social well-being of individuals and communities, experts estimate that the current wave of unemployment could raise global suicides by thousands (14, 15). Among dental professionals in particular, recent research in Italy has discovered a positive correlation between the COVID-19 shutdowns and increased levels of anxiety and career concerns (16). We sought to discover if there were merely a correlation or a substantial causative effect between unemployment among dental specialty workers and future COVID-19 vaccine acceptance. Dentistry is unique in that it is a private-sector workforce which, unlike most healthcare fields, was forced to discontinue non-essential operations in Israel, as well as in other countries, during the pandemic (17). This characteristic can allow extrapolations from questionnaires distributed to dentistry or dentistry-auxiliary employees regarding unemployment and vaccine willingness.

In the current article we hypothesized a possible explanation for low acceptance rates for the novel SARS-CoV-2 vaccine among dentistry workers. As shown here, unchanged employment status significantly correlates with reduced compliance to the novel SARS-CoV-2 vaccine. Although countries worldwide are attempting to manage the current crisis, the presented trends among the working population should alert governments and organizations about anticipated vaccination rates among their residents. Furthermore, the upcoming winter could present a colossal burden to the healthcare system due to the expected seasonality of respiratory viral infections, potentially once again leading governments to use lockdowns once again as tools of curbing the spread of COVID-19 (18). Historical perspectives indicate pandemic outbreaks occur in 10–50 years intervals, suggesting that a majority of the population will likely experience another pandemic in their lifetimes (19). In light of comparisons between the effects of the SARS-CoV-2 virus and the seasonal influenza virus, governments must pay close attention to employment as a factor in future campaigns to encourage vaccination.

Our results demonstrate a positive correlation between unemployment rate and willingness to receive a COVID-19 vaccine. Despite the small sample size of 506 respondents, the *r* = 0.9414 and low *p-*value indicate a significant and demonstrable correlation. Our research presents a unique influencing factor on vaccine hesitancy: employment rates. The results of over 500 dental and dentistry-adjacent respondents do indicate a positive correlation between the vaccine acceptance and unemployment. Our research furthers existing investigations into common factors between vaccine-hesitant individuals and identifies a statistically significant relationship between employment status in the current crisis and SARS-CoV-2 vaccine acceptance. High or low unemployment could be another examining tool to determine which professions and communities are at risk of vaccination hesitancy. While our paper excluded racial, ethnic, and religious characteristics and examines only self-identified Israeli dental professionals, stratification based on specific demographical criteria warrants future investigation. Further exploration of the attitudes of oral healthcare professionals globally toward a COVID-19 vaccine would likely be of broad research interest, as well. Moreover, our findings add to a growing body of research on vaccination among oral healthcare professionals. Close observation of professions with high rates of employment could potentially lead to early interventional, educational campaigns regarding the benefit of vaccines not only for the individual, but also for communities at large.

Limitations of our research include that our investigation is within a single country and that broader occupations were not included. Further research can and should delineate whether physicians working in private clinics, which may have been ordered to shut down except for essential services, vs. physicians in hospitals which were not shuttered, have differences in COVID-19 vaccine acceptance rates. Additionally, the explanations for differing vaccine acceptance rates among even one class of profession, such as physicians or dentists, must be clarified.

## Data Availability Statement

The original contributions presented in the study are included in the article/Supplementary Material, further inquiries can be directed to the corresponding author/s.

## Ethics Statement

The study design and protocol were approved by the Research Ethics Committee of Galilee Medical Center and the web-based survey followed the American Association for Public Opinion Research (AAPOR) reporting guidelines.

## Author Contributions

AZ, AAD, TS, TH, NM, NE, DR, FK, ES, HM, and SS were involved in the development of this manuscript and gave final approval before submission. All authors attest they meet the ICMJE criteria for authorship.

## Conflict of Interest

The authors declare that the research was conducted in the absence of any commercial or financial relationships that could be construed as a potential conflict of interest.
